# Supplementary material for: Zero-shot performance analysis of large language models in sumrate maximization
Source: PLoS One. 2025 Aug 4;20(8):e0329674. doi: 10.1371/journal.pone.0329674 (PMC12321130; doi:10.1371/journal.pone.0329674)
Supplement: Attachment — Submitted filename: Response to Reviewers.pdf [file pone.0329674.s001.pdf]

#	Reviewer	Comment	Resolution	Manuscript Reference
1	Reviewer 1	The insights are from the testing of LLM. The paper is more like a technical report of LLM in networking. More insights should be added. For example, how can we improve the LLM to meet the requirement of network optimziation tasks.	We appreciate the reviewer's concern. We have added a separate subsection - "Improving LLM for Network Optimization". In this section, we have added our insight on how to improve LLM to meet network optimization task requirements	**Page 8, Line 251-258**
2	Reviewer 1	Moreover, except directly using LLM, is there any other way to use LLM to enhance the network?	In Section 4.3, we proposed an idea about using LLM as an intermediary. It can provide outputs to help other algorithms in achieving better performance in tasks like Sumrate Maximization.	**Page 8, Line 259-264**
3	Reviewer 2	As the problem is solved using GPT, and the proposed solution is sub-optimal, the reviewer is not sure if the LLM is able to propose a good enough solution. Even though using LLM for sum-rate optimization is unique, but the results are not good enough to ensure that LLM is really useful for all such tasks.	We thank the reviewer for pointing this out. LLMs can be used to make quick decisions without having prior algorithmic knowledge. This was unclear earlier and now we have specified it in our manuscript.	**Page 7, Line 210-212**
4	Reviewer 2	Promt engineering is not explored well for creating algorithms and it needs extensive study and comparison with existing algorithms and show comparable results to establish that LLM can be used for sumrate maximization in wireless communication.	Our prompt has evolved through multiple iterations while judging from the outputs of the LLMs. We extensively analyzed the outputs to understand the need of the models and we provided more information to obtain our desired result. We have now added the changes made to each iteration while developing our prompt.	**Page 3-4, Line 115-126**
5	Reviewer 2	The system model is not present, which make it difficult to follow.	Section 2 - Methodology has subsections which are the steps taken by us for conducting our experiment. This is now specified in the manuscript.	**Page 3, Line 104-105**
6	Reviewer 2	Table 3 does not have explanation.	We are grateful to the reviewer for identifying this as it will definitely help improve the quality of the manuscript. We have added a proper explanation to the table to assist the readers.	**Page 5, Line 166-170**
7	Reviewer 2	Fig 2 needs explanation.	A proper explanation of the figure with all the features is currently added. A summary of the main gist of the figure is also added in one sentence.	**Page 7, Line 199-202**
