# Supplementary material for: Zero-shot performance analysis of large language models in sumrate maximization
Source: PLoS One. 2025 Aug 4;20(8):e0329674. doi: 10.1371/journal.pone.0329674 (PMC12321130; doi:10.1371/journal.pone.0329674)
Supplement: Attachment — Submitted filename: Response_to_Reviewers_auresp_2.pdf [file pone.0329674.s002.pdf]

#	Reviewer	Comment	Resolution	Manuscript Reference
1	Reviewer 1	Most my previous comments are addressed. This paper can provide some insights for the applications of LLM in the network issues. However, one important things is that most refs discussed in this paper is about the LLM from the AI perspective. Considering that recently there are many works that use LLM in the network optimization, e.g., together with reinforcement learning or RAG, it is necessary to discuss these papers to provide the readers a detailed background.	We appreciate the reviewer's concern. We have discussed another manuscript in our Related Works section which is focused on this particular suggestion.	**Page 3, Line 96-97**
2	Reviewer 2	Specify the hardware and software environment used for the experiment	We have added another subsection (Section 3.1.1) titled "Hardware and Software Environments" under the Section Experimental Setup where we discussed every experimental environments used.	**Page 5, Line 159-166**
3	Reviewer 2	It would be helpful if the authors provided insight into the parameters used for the OpenAI ChatCompletion to generate the response, e.g., max tokens, conversation: system, user, etc.	We thank the reviewer for pointing this out. We have now added another subsection, "Parametric Values of the LLMs," where we discuss these in detail for the readers.	**Page 5, Line 167-172**
4	Reviewer 2	Replace the generic phrase “In the result comparison graph” used in section 2.3 with an explicit pointer (e.g., “As shown in Figure 3…”). To ensure the graph refrenced is understood by the readers.	We corrected this cosmetic issue and accepted the reviewers suggestion.	**Page 5, Line 152**
