# Supplementary material for: Zero-shot performance analysis of large language models in sumrate maximization
Source: PLoS One. 2025 Aug 4;20(8):e0329674. doi: 10.1371/journal.pone.0329674 (PMC12321130; doi:10.1371/journal.pone.0329674)
Supplement: Attachment — Submitted filename: Response_to_Reviewers_auresp_3.pdf [file pone.0329674.s003.pdf]

#	Reviewer	Comment	Resolution	Manuscript Reference
1	Reviewer 3	Although the introduction is brief and to the point, it would be beneficial to include a short explanation of what sum-rate maximization entails, particularly for readers who may not be specialists in this area. This addition would enhance accessibility for a broader audience.	We appreciate the reviewer's concern. We have discussed about sumrate maximization in our introduction for the readers to have a clearer idea about the topic.	**Page 2, Line 26-27**
2	Reviewer 3	The manuscript stated a valid concerns regarding the limitations of large language models (LLMs) in reasoning in the related works section (line 59 – 63) of the manusctipt. However, recent advancements have introduced techniques such as chain-of-thought prompting, and scratchpad reasoning which have significantly enhanced the performance of LLMs in structured reasoning tasks. Instead of broadly stating that LLMs struggle with reasoning, I recommend refining this point to reflect that while current models have improved, their reasoning abilities can still be fragile or dependent on context.	We have discussed about chain-of-thought prompting and scratchpad reasoning in our manuscript taking into account the reviewer's suggestion. We thank the reviewers for this valuable feedback.	**Page 2, Line 62-63**
3	Reviewer 3	Additionally, the manuscript employs GPT-3.5-Turbo, which is now considered a relatively outdated version of OpenAI's models. It would be helpful to explain the rationale behind selecting this version. If possible, consider rerunning the experiments using more recent models with stronger reasoning capabilities, such as GPT-4o or o3-mini, as they may yield improved results and strengthen your conclusions.	We thank the reviewer for pointing this out. The scope of the work, when submitted, was with GPT 3.0 and we are certain that newer models might outperform this model. But the research is regarding the then version of GPT and if that shows significant performance boost (which it does), we can conclude that newer models would follow the trend.	**Page 7, Line 222-223**
